# Supplementary material for: Joining a group diverts regret and responsibility away from the individual
Source: Proc Biol Sci. 2020 Mar 11;287(1922):20192251. doi: 10.1098/rspb.2019.2251 (PMC7126069; doi:10.1098/rspb.2019.2251)
Supplement: All supplementary materials [file rspb20192251supp1.docx]

SUPPLEMENTARY MATERIALS

El Zein & Bahrami

Joining a group diverts regret and responsibility away from the individual

Proceedings of the Royal Society B

Article doi: *10.1098/rspb.2019-2251*

| **Supplementary table 1 - Lottery structure** | | | | | | | | | | | | | | | | |
| --- | --- | --- | --- | --- | --- | --- | --- | --- | --- | --- | --- | --- | --- | --- | --- | --- |
| Lottery number | x1 | p | y1 | 1-p | x2 | q | y2 | 1-q | ΔEV | ΔAR | Group choice EXP1 | Exp 2 % Group Choice Lottery1 | Exp 2 % Group Choice Lottery2 | Exp 2 %Individual Choice Lottery 1 | Exp 2 %Individual Choice Lottery 2 | Exp 2 mean % Lottery 1 reference group |
| 1 | 200 | 0.8 | 50 | 0.2 | 200 | 0.2 | 50 | 0.8 | 90 | 0 | 1 | 98.4 | 1.6 | 90.9 | 9.1 | 88.7 |
| 2 | 200 | 0.2 | 50 | 0.8 | 200 | 0.5 | 50 | 0.5 | -45 | 0 | 2 | 2.1 | 97.9 | 13.6 | 86.4 | 13.8 |
| 3 | 200 | 0.5 | -50 | 0.5 | 50 | 0.5 | -50 | 0.5 | 75 | 150 | 1 | 98.7 | 1.3 | 87.2 | 12.8 | 87.2 |
| 4 | 50 | 0.8 | -50 | 0.2 | 200 | 0.2 | -200 | 0.8 | 150 | 0 | 2 | 96.6 | 3.4 | 87.1 | 12.9 | 83 |
| 5 | 50 | 0.2 | -50 | 0.8 | 200 | 0.5 | 50 | 0.5 | -155 | -250 | 2 | 0 | 100 | 6.4 | 93.6 | 7.3 |
| 6 | 200 | 0.5 | -200 | 0.5 | 200 | 0.8 | 50 | 0.2 | -170 | -250 | 2 | 2.9 | 97.1 | 13.4 | 86.6 | 15.1 |
| 7 | 50 | 0.8 | -200 | 0.2 | 200 | 0.5 | -50 | 0.5 | -75 | -300 | 2 | 9.8 | 90.2 | 26.5 | 73.5 | 24 |
| 8 | 200 | 0.5 | 50 | 0.5 | 50 | 0.5 | -50 | 0.5 | 125 | 250 | 1 | 99.6 | 0.4 | 92.4 | 7.6 | 90 |
| 9 | 50 | 0.8 | -200 | 0.2 | 200 | 0.8 | -200 | 0.2 | -120 | -150 | 2 | 4.6 | 95.4 | 14.7 | 85.3 | 16.6 |
| 10 | 50 | 0.2 | -50 | 0.8 | -50 | 0.5 | -200 | 0.5 | 95 | 250 | 1 | 76.9 | 23.1 | 69.3 | 30.7 | 62.9 |
| 11 | 50 | 0.5 | -50 | 0.5 | 50 | 0.5 | -200 | 0.5 | 75 | 150 | 1 | 95.6 | 4.4 | 82.2 | 17.8 | 79.3 |
| 12 | 50 | 0.2 | -200 | 0.8 | 50 | 0.2 | -50 | 0.8 | -120 | -150 | 2 | 9.7 | 90.3 | 21 | 79 | 21.4 |
| 13 | 200 | 0.5 | -200 | 0.5 | 50 | 0.5 | -200 | 0.5 | 75 | 150 | 1 | 97 | 3 | 86.1 | 13.9 | 85.2 |
| 14 | 50 | 0.2 | -200 | 0.8 | 50 | 0.8 | -200 | 0.2 | -150 | 0 | 2 | 0.4 | 99.6 | 9.3 | 90.7 | 7.1 |
| 15 | 200 | 0.8 | -200 | 0.2 | 50 | 0.2 | -200 | 0.8 | 270 | 150 | 1 | 100 | 0 | 93.6 | 6.4 | 93.2 |
| 16 | 50 | 0.5 | -200 | 0.5 | 50 | 0.8 | -200 | 0.2 | -75 | 0 | 2 | 4.8 | 95.2 | 16.9 | 83.1 | 20.8 |
| 17 | 200 | 0.5 | -200 | 0.5 | 50 | 0.8 | -50 | 0.2 | -30 | 0 | 1 | 65 | 35 | 43.5 | 56.5 | 61.1 |
| 18 | 200 | 0.8 | -50 | 0.2 | 200 | 0.5 | 50 | 0.5 | 25 | -100 | 1 | 73.9 | 26.1 | 52.9 | 47.1 | 61.5 |
| 19 | -50 | 0.2 | -200 | 0.8 | 200 | 0.2 | -200 | 0.8 | -50 | -250 | 2 | 4.3 | 95.7 | 19.1 | 80.9 | 18.7 |
| 20 | -50 | 0.2 | -200 | 0.8 | 200 | 0.5 | -200 | 0.5 | -170 | -250 | 2 | 0.4 | 99.6 | 7 | 93 | 4.8 |
| 21 | 200 | 0.8 | 50 | 0.2 | 200 | 0.5 | -50 | 0.5 | 95 | 100 | 1 | 98.7 | 1.3 | 88.1 | 11.9 | 87.8 |
| 22 | 200 | 0.2 | -200 | 0.8 | 50 | 0.2 | -50 | 0.8 | -90 | 0 | 1 | 54.6 | 45.4 | 44.1 | 55.9 | 50.8 |
| 23 | 200 | 0.8 | -50 | 0.2 | 50 | 0.8 | -50 | 0.2 | 120 | 150 | 1 | 100 | 0 | 88.5 | 11.5 | 88.9 |
| 24 | -50 | 0.8 | -200 | 0.2 | 200 | 0.2 | -200 | 0.8 | 40 | -250 | 1 | 44.1 | 55.9 | 49 | 51 | 47.3 |
| 25 | -50 | 0.5 | -200 | 0.5 | 200 | 0.8 | -200 | 0.2 | -245 | -250 | 2 | 0 | 100 | 6.1 | 93.9 | 6.8 |
| 26 | -50 | 0.8 | -200 | 0.2 | 200 | 0.8 | -50 | 0.2 | -230 | -400 | 2 | 0.4 | 99.6 | 7.8 | 92.2 | 10.1 |
| 27 | -50 | 0.2 | -200 | 0.8 | 50 | 0.8 | -200 | 0.2 | -170 | -100 | 2 | 0 | 100 | 5.9 | 94.1 | 4.9 |
| 28 | -50 | 0.8 | -200 | 0.2 | -50 | 0.5 | -200 | 0.5 | 45 | 0 | 1 | 81.6 | 18.4 | 78.5 | 21.5 | 68.1 |
| 29 | 50 | 0.5 | -50 | 0.5 | 50 | 0.2 | -200 | 0.8 | 150 | 150 | 1 | 99.2 | 0.8 | 91.4 | 8.6 | 88.7 |
| 30 | 200 | 0.8 | -200 | 0.2 | -50 | 0.2 | -200 | 0.8 | 290 | 250 | 1 | 99.6 | 0.4 | 96.3 | 3.7 | 91.4 |
| 31 | 200 | 0.5 | -50 | 0.5 | -50 | 0.5 | -200 | 0.5 | 200 | 400 | 1 | 100 | 0 | 93 | 7 | 92.2 |
| 32 | -50 | 0.8 | -200 | 0.2 | 200 | 0.2 | -50 | 0.8 | -80 | -400 | 2 | 29.8 | 70.2 | 32.3 | 67.7 | 40.6 |
| 33 | 200 | 0.2 | -50 | 0.8 | 200 | 0.8 | 50 | 0.2 | -170 | -100 | 2 | 0.4 | 99.6 | 5.7 | 94.3 | 7.7 |
| 34 | 200 | 0.8 | -50 | 0.2 | 50 | 0.8 | -50 | 0.2 | 120 | 150 | 1 | 98.7 | 1.3 | 91.3 | 8.7 | 89.8 |
| 35 | 50 | 0.2 | -50 | 0.8 | 200 | 0.5 | 50 | 0.5 | -155 | -250 | 2 | 0.4 | 99.6 | 7 | 93 | 6.9 |
| 36 | 200 | 0.5 | 50 | 0.5 | 50 | 0.2 | -50 | 0.8 | 155 | 250 | 1 | 100 | 0 | 96.3 | 3.7 | 97.6 |
| 37 | 200 | 0.5 | 50 | 0.5 | 200 | 0.8 | -50 | 0.2 | -25 | 100 | 2 | 42.9 | 57.1 | 51.7 | 48.3 | 45 |
| 38 | 50 | 0.8 | -50 | 0.2 | 200 | 0.8 | -50 | 0.2 | -120 | -150 | 2 | 1.7 | 98.3 | 10.6 | 89.4 | 9 |
| 39 | 50 | 0.5 | -50 | 0.5 | 50 | 0.5 | -200 | 0.5 | 75 | 150 | 1 | 95.5 | 4.5 | 82.9 | 17.1 | 79.9 |
| 40 | 200 | 0.8 | 50 | 0.2 | 200 | 0.5 | -50 | 0.5 | 95 | 100 | 1 | 99.6 | 0.4 | 89.3 | 10.7 | 90.6 |
| 41 | 200 | 0.5 | -50 | 0.5 | 50 | 0.8 | -200 | 0.2 | 75 | 300 | 1 | 87.4 | 12.6 | 72.7 | 27.3 | 73 |
| 42 | 50 | 0.5 | -200 | 0.5 | 200 | 0.5 | -200 | 0.5 | -75 | -150 | 2 | 2.6 | 97.4 | 12.1 | 87.9 | 17.1 |
| 43 | 50 | 0.8 | -200 | 0.2 | 200 | 0.8 | -200 | 0.2 | -120 | -150 | 2 | 3.4 | 96.6 | 15.2 | 84.8 | 18.6 |
| 44 | 200 | 0.5 | -200 | 0.5 | -50 | 0.2 | -200 | 0.8 | 170 | 250 | 1 | 99.2 | 0.8 | 94.9 | 5.1 | 94 |
| 45 | 200 | 0.2 | -200 | 0.8 | -50 | 0.8 | -200 | 0.2 | -40 | 250 | 1 | 61.8 | 38.2 | 53.4 | 46.6 | 58 |
| 46 | 200 | 0.8 | -200 | 0.2 | 200 | 0.5 | -50 | 0.5 | 45 | -150 | 1 | 83 | 17 | 63.5 | 36.5 | 70.3 |
| 47 | 200 | 0.5 | -50 | 0.5 | 200 | 0.8 | -200 | 0.2 | -45 | 150 | 2 | 7.5 | 92.5 | 35.7 | 64.3 | 21.1 |
| 48 | 200 | 0.5 | -200 | 0.5 | 200 | 0.2 | -200 | 0.8 | 120 | 0 | 1 | 99.6 | 0.4 | 91.2 | 8.8 | 88.9 |

Description of table variables: p and q are the probabilities of getting the best outcomes, X1 (lottery 1) and X2 (lottery 2), respectively. Y1 and Y2 represent the worst outcomes for lotteries 1 and 2, respectively. ΔEV corresponds to the difference in expected value between lottery 1 and lottery 2. ΔAR represents the difference in anticipated regret between the two lotteries. Group choice Exp1 reports for each lottery pair the gamble chosen by the fixed pilot group. Exp 2 % Group Choice Lottery1 reports the percentage of times where Lottery 1 was chosen by the randomly drawn group; Exp 2 % Group Choice Lottery2 is the same as the previous column for Lottery 2; Exp 2 %Individual Choice Lottery 1 reports the percentage of **individual** choices of Lottery 1; Exp 2 %Individual Choice Lottery 2 is the same as the previous column for Lottery 2. Exp 2 mean % Lottery 1 reference group reports the % choice of the Lottery 1 for the Reference group (i.e., the 4 randomly drawn previous participants, without the participant’s choice).

**Statistical details of mixed models**

**Supplementary table 2 - Experiment 1**

| Group choice | | | | | | |
| --- | --- | --- | --- | --- | --- | --- |
| Regressors | Parameter estimate | Standard error of estimate | 95% Confidence interval | Z value | P value | Number of observations |
| Previous condition (t-1) | 4.77 | 0.53 | [3.64, 6.08], | 8.96 | <.001 | 4268 |
| Previous condition*Valence (t-1) | 0.54 | 0.11 | [0.32, 0.77] | 4.79 | <.001 | 4268 |
| Valence alone (t-1) | -0.258 | 0.083 | [-0.63, -0.16] | 3.114 | 0.00185 | 2419 |
| Valence group (t-1) | 0.28 | 0.079 | [0.12, 0.46] | 3.57 | <.001 | 1849 |
| Previous conditionXMagnitude (t-1) | 0.19 | 0.14 | [-0.092, 0.47] | 1.34 | 0.17 | 4268 |
| Magnitude Alone (t-1) | -0.13 | 0.102 | [-0.35, 0.067] | -1.35 | 0.17 | 2419 |
| Magnitude Group | 0.056 | 0.099 | [-0.14, 0.26] | 0.56 | 0.56 | 1849 |
| Group Status (t-1) | -0.47 | 0.18 | [-0.84, 0.103] | -2.55 | 0.0105 | 1849 |
| Group Status*Valence(t-1) | 0.37 | 0.18 | [0.0088, 0.73] | 2.04 | 0.0413 | 1849 |
| Valence minority (t-1) | 0.49 | 0.14 | [-0.071, 0.69] | 3.32 | <.001 | 407 |
| Valence majority (t-1) | 0.16 | 0.09 | [-0.29, 0.18] | 1.644 | 0.10 | 1442 |
| Lottery choice | | | | | | |
| Expected value ΔEV (t) | 2.15 | 0.086 | [1.98, 2.33] | 25.03 | <.001 | 5799 |
| Anticipated regret ΔAR(t) | 0.15 | 0.067 | [0.020, 0.28] | 2.26 | 0.0233 | 5799 |
| Current condition(t) X ΔEV(t) | -0.62 | 0.12 | [-0.86, -0.38] | -5.14 | <.001 | 5799 |
| Current condition(t) X ΔAR(t) | -0.017 | 0.0982 | [-0.21, 0.17] | -0.175 | 0.8612 | 5799 |
| ΔEV alone(t) | 2.158 | 0.086 | [1.98, 2.33], | 25.029 | <0.001 | 3326 |
| ΔEV group(t) | 1.538 | 0.0842 | [1.37, 1.70] | 18.254 | <0.001 | 2473 |
| ΔAR alone(t) | 0.152 | 0.067 | [0.02, 0.28] | 2.269 | 0.0232 | 3326 |
| ΔAR group(t) | 0.13 | 0.071 | [0.0056, 0.27] | 1.88 | 0.059 | 2473 |
| Previous condition(t-1) X Valence (t-1) X ΔEV(t) | -0.48 | 0.16 | [-0.69, -0.15] | -2.88 | 0.0039 | 4268 |
| Valence (t-1) X ΔEV(t) alone | 0.30 | 0.107 | [0.091, 0.51] | 2.835 | 0.00458 | 2419 |
| Valence (t-1) X ΔEV(t) group | -0.172 | 0.127 | [-0.42, 0.07] | -1.35 | 0.177 | 1849 |
| ΔEV(t) alone negative | 1.63 | 0.17 | [1.29, 1.99] | 9.21 | <.001 | 627 |
| ΔEV(t) alone positive | 2.26 | 0.12 | [2.02, 2.51] | 18.55 | <.001 | 1792 |
| Group status(t-1) X Valence(t-1) X ΔEV(t) | -0.56 | 0.29 | [-1.15, 0.00017] | -1.91 | 0.0561 | 1849 |
| Valence (t-1) X ΔEV(t) minority | -0.57 | 0.25 | [-1.10, -0.095] | -2.23 | 0.025, | 407 |
| ΔEV(t) after negative minority | 2.44 | 0.44 | [1.62, 3.38] | 5.493 | <.001 | 128 |
| ΔEV(t) after positive minority | 1.43 | 0.25 | [0.93,1.96] | 5.693 | <.001 | 279 |

**Supplementary table 3 - Experiment 2**

| Group choice | | | | | | |
| --- | --- | --- | --- | --- | --- | --- |
| Regressors | Parameter estimate | Standard error of estimate | 95% Confidence interval | Z value | P value | Number of observations |
| Previous condition (t-1) | 5.30 | 0.23 | [1.15, 1.39] | 21.35 | <.001 | 17178 |
| Previous condition*Valence (t-1) | 0.49 | 0.061 | [0.32, 0.55] | 8.00 | <.001 | 17178 |
| Valence alone (t-1) | -0.17 | 0.044 | [-0.26, -0.085] | -3.91 | <.001 | 8861 |
| Valence group (t-1) | 0.31 | 0.042 | [0.23, 0.40] | 7.41 | <.001 | 8317 |
| Previous conditionXMagnitude (t-1) | 0.15 | 0.078 | [-0.0029, 0.31] | 1.96 | 0.04 | 17178 |
| Magnitude Alone (t-1) | -0.094 | 0.054 | [-0.20, 0.013] | -1.74 | 0.08 | 8861 |
| Magnitude Group | 0.054 | 0.056 | [-0.061, 0.16] | 0.95 | 0.33 | 8317 |
| Group Status (t-1) | -0.43 | 0.103 | [-0.64, -0.22] | -4.15 | <.001 | 8317 |
| Group Status*Valence(t-1) | 0.11 | 0.10 | [-0.089, 0.32] | 1.14 | 0.25 | 8317 |
| Valence minority (t-1) | 0.34 | 0.089 | [0.16, 0.52] | 3.82 | <.001 | 6944 |
| Valence majority (t-1) | 0.27 | 0.050 | [0.17, 0.37] | 5.57 | <.001 | 1373 |
| Lottery choice | | | | | | |
| Expected value ΔEV (t) | 2.0 | 0.04 | [1.98, 2.16] | 46.27 | <.001 | 23247 |
| Anticipated regret ΔAR(t) | 0.26 | 0.035 | [0.19, 0.33] | 7.42 | <.001 | 23247 |
| Current condition(t) X ΔEV(t) | -0.33 | 0.061 | [-0.45, -0.21] | -5.45 | <.001 | 23247 |
| Current condition(t) X ΔAR(t) | -0.12 | 0.049 | [-0.22, -0.025] | -2.48 | 0.0131 | 23247 |
| ΔEV alone(t) | 2.07 | 0.044 | [1.98, 2.16] | 46.27 | <.001 | 12087 |
| ΔEV group(t) | 1.73 | 0.041 | [1.65, 1.82] | 41.52 | <.001 | 11160 |
| ΔAR alone(t) | 0.26 | 0.035 | [0.19, 0.33] | 7.424 | <.001 | 12087 |
| ΔAR group(t) | 0.14 | 0.034 | [0.072, 0.21] | 4.048 | <.001 | 11160 |
| Previous condition(t-1) X Valence (t-1) X ΔEV(t) | -0.19 | 0.082 | [-0.35, -0.031] | -2.33 | .019 | 17178 |
| Valence (t-1) X ΔEV(t) alone | 0.238 | 0.056 | [0.12, 0.349] | 4.186 | <.001 | 8861 |
| Valence (t-1) X ΔEV(t) group | 0.044 | 0.059 | [-0.072, 0.16] | 0.75 | 0.44 | 8317 |
| ΔEV(t) alone negative | 1.78 | 0.095 | [1.59, 1.98] | 18.79 | <.001 | 2317 |
| ΔEV(t) alone positive | 2.25 | 0.064 | [2.12, 2.39] | 34.89 | <.001 | 6544 |
| Group status(t-1) X Valence(t-1) X ΔEV(t) | -0.31 | 0.14 | [-0.59, -0.035] | -2.17 | 0.029 | 8317 |
| Valence (t-1) X ΔEV(t) minority | -0.208 | 0.127 | [0.054, 0.77] | -1.63 | 0.102 | 1373 |
| ΔEV(t) after negative minority | 1.96 | 0.23 | [1.47, 2.55] | 8.33 | <.001 | 430 |
| ΔEV(t) after positive minority | 1.40 | 0.12 | [1.14, 1.67] | 10.91 | <.001 | 843 |

**Supplementary table 4 –** Meta-analytic results across Experiments 1 and 2

| Group choice | | | | | |
| --- | --- | --- | --- | --- | --- |
| Regressors | Parameter estimate | Standard error of estimate | 95% Confidence interval | Z value | P value |
| Previous condition (t-1) | 5.21 | 0.21 | [4.78, 5.64] | 22.7 | <.001 |
| Previous condition*Valence (t-1) | 0.50 | 0.05 | [0.39, 060] | 8.31 | <.001 |
| Valence alone (t-1) | -0.19 | 0.03 | [-0.26, -0.11] | -4.9 | <.001 |
| Valence group (t-1) | 0.30 | 0.037 | [0.23,0.38] | 8.22 | <.001 |
| Previous conditionXMagnitude (t-1) | 0.16 | 0.06 | [0.027,0.29] | 2.36 | 0.01 |
| Magnitude Alone (t-1) | -0.104 | 0.047 | [-0.19, -0.01] | -2.17 | 0.02 |
| Magnitude Group | 0.054 | 0.049 | [-0.04, 0.15] | 1.11 | 0.26 |
| Group Status (t-1) | -0.44 | 0.09 | [-0.61, -0.26] | -4.87 | <.001 |
| Group Status*Valence(t-1) | 0.19 | 0.11 | [-0.03, 0.43] | 1.67 | 0.09 |
| Valence minority (t-1) | 0.38 | 0.076 | [0.23, 0.53] | 4.99 | <.001 |
| Valence majority (t-1) | 0.24 | 0.051 | [0.14, 0.34] | 4.84 | <.001 |
| Lottery choice | | | | | |
| Expected value ΔEV (t) | 2.09 | 0.039 | [2.01, 2.17] | 52.59 | <.001 |
| Anticipated regret ΔAR(t) | 0.22 | 0.055 | [0.12, 0.33] | 4.14 | <.001 |
| Current condition(t) X ΔEV(t) | -0.45 | 0.14 | [-0.73, -0.18] | -3.24 | 0.0012 |
| Current condition(t) X ΔAR(t) | -0.10 | .044 | [-0.18, -0.014] | -2.29 | 0.0218 |
| ΔEV alone(t) | 2.09 | 0.03 | [2.01, 2.16] | 52.59 | <.001 |
| ΔEV group(t) | 1.65 | 0.099 | [1.45, 1.84] | 16.63 | <.001 |
| ΔAR alone(t) | 0.22 | 0.053 | [0.12, 0.32] | 4.15 | <.001 |
| ΔAR group(t) | 0.139 | 0.031 | [0.078, 0.20] | 4.46 | <.001 |
| Previous condition(t-1) X Valence (t-1) X ΔEV(t) | -0.30 | 0.14 | [-0.57, -0.025] | -2.14 | 0.0322 |
| Valence (t-1) X ΔEV(t) alone | 0.25 | 0.050 | [0.15, 0.35] | 5.02 | <.001 |
| Valence (t-1) X ΔEV(t) group | -0.0343 | 0.10 | [-0.23, 0.17] | -0.32 | 0.743 |
| ΔEV(t) alone negative | 1.75 | 0.083 | [1.58, 1.916] | 20.92 | <.001 |
| ΔEV(t) alone positive | 2.25 | 0.057 | [2.14, 2.37] | 39.5 | <.001 |
| Group status(t-1) X Valence(t-1) X ΔEV(t) | -0.36 | 0.128 | [-0.61, -0.107] | -2.79 | 0.0052 |
| Valence (t-1) X ΔEV(t) minority | -0.32 | 0.17 | [-0.66, 0.011] | -1.89 | 0.0582 |
| ΔEV(t) after negative minority | 2.069 | 0.208 | [1.66, 2.47] | 9.93 | <.001 |
| ΔEV(t) after positive minority | 1.40 | 0.11 | [1.18, 1.63] | 12.3 | <.001 |

**Supplementary table 5**

Meta-analytic results across both experiment after removing participants who consistently played alone or in group (Leaving 65/125 participants in Exp 1 and 227/496 participants in Exp 2).

| Regressors | Parameter estimate | Standard error of estimate | 95% Confidence interval | Z value | P value |
| --- | --- | --- | --- | --- | --- |
| Previous condition | 1.05 | 0.43 | [0.20, 1.91] | 2.42 | 0.0155 |
| Previous conditionXValence | 0.48 | 0.055 | [0.37, 0.59] | 8.7668 | <.001 |
| Valence alone | -0.18 | 0.039 | [-0.26, -0.10] | -4.650 | <.001 |
| Valence group | 0.29 | 0.038 | [0.22, 0.37] | 7.71 | <.001 |
| Previous conditionXMagnitude | 0.14 | 0.07 | [0.012, 0.28] | 2.12 | 0.033 |
| Magnitude Alone | -0.09 | 0.048 | [-0.18, 0.004] | -1.859 | 0.0630 |
| Magnitude Group | 0.056 | 0.05 | [-0.042, 0.15] | 1.11 | 0.264 |
| Group Status | -0.36 | 0.093 | [-0.54, -0.18] | -3.919 | <.001 |
| Group StatusXValence | 0.14 | 0.091 | [-0.03, 0.32] | 1.62 | 0.1038 |
| Minority | 0.36 | 0.076 | [0.21, 0.51] | 4.70 | <.001 |
| Majority | 0.24 | 0.045 | [0.16, 0.33] | 5.50 | <.001 |

**Supplementary table 6 - Experiment 2b**

Experiment 2b is a previous version of Experiment 2. We realized during the review process that only half of the lottery structures (24 out of 48 total from Coricelli et al., 2005) were actually presented to each participant, due to an error in the randomization script of the experiment. The error does not falsify the analyses done, as it only reduced the variety in the lotteries shown, we therefore report these results for completeness.

| **Group choice** | | | | | | |
| --- | --- | --- | --- | --- | --- | --- |
| **Regressors** | **Parameter estimate** | **Standard error of estimate** | **95% Confidence interval** | **Z value** | **P value** | **Number of observations** |
| Previous condition (t-1) | 4.67 | 0.22 | [4.15, 5.22] | 20.83 | <.001 | 14425 |
| Previous conditionXValence (t-1) | 0.28 | 0.06 | [0.15, 0.407] | 4.52 | <.001 | 14425 |
| Valence alone (t-1) | -0.0948 | 0.045 | [-0.28, -0.06] | -2.07 | 0.0378 | 7397 |
| Valence group (t-1) | 0.171 | 0.043 | [0.13, 0.35] | 3.91 | <.001 | 7028 |
| Previous conditionXMagnitude (t-1) | 0.14 | 0.075 | [-0.01, 0.30] | 1.94 | 0.05 | 14425 |
| Magnitude Alone (t-1) | -0.039 | 0.051 | [-0.14, 0.066] | -0.76 | 0.44 | 7397 |
| Magnitude Group (t-1) | 0.11 | 0.056 | [-0.002, 0.22] | 1.97 | 0.048 | 7028 |
| Previous conditionXMagnitude negative* | 0.384 | 0.137 | [0.10, 0.66] | 2.78 | 0.00529 | 3935 |
| Magnitude negative alone* | -0.25 | 0.091 | [-0.43, -0.068] | -2.77 | 0.00557 | 2281 |
| Magnitude negative group* | 0.16 | 0.09 | [-0.07, 0.34] | 1.64 | 0.10 | 1654 |
| Group Status (t-1) | -0.39 | 0.096 | [-0.59, -0.19] | -4.12 | <.001 | 7028 |
| Group StatusXValence (t-1) | 0.060 | 0.094 | [-0.13, 0.25] | 0.643 | 0.520 | 7028 |
| Valence minority (t-1) | 0.15 | 0.077 | [0.00095, 0.31] | 2.035 | 0.0418 | 1818 |
| Valence majority (t-1) | 0.16 | 0.055 | [0.055, 0.27] | 3.02 | 0.00247 | 5210 |
| **Lottery choice** | | | | | | |
| Expected value ΔEV (t) | 1.589 | 0.044 | [1.50, 1.67] | 36.007 | <.001 | 19563 |
| Anticipated regret ΔAR(t) | 0.1413 | 0.037 | [0.067, 0.21] | 3.748 | <.001 | 19563 |
| Current condition(t) X ΔEV(t) | -0.485 | 0.05927 | [-0.60, -0.36] | -8.18 | <.001 | 19563 |
| Current condition(t) X ΔAR(t) | -0.147 | 0.05227 | [-0.25, -0.044] | -2.82 | 0.0047 | 19563 |
| ΔEV alone(t) | 1.59 | 0.044 | [1.50, 1.68] | 35.981 | <0.001 | 10070 |
| ΔEV group(t) | 1.10 | 0.039 | [1.02, 1.18] | 27.86 | <0.001 | 9493 |
| ΔAR alone(t) | 0.14 | 0.037 | [0.066, 0.21] | 3.737 | <0.001 | 10070 |
| ΔAR group(t) | 0.00478 | 0.036 | [-0.076, 0.066] | -0.133 | 0.894 | 9493 |
| Previous condition(t-1) X Valence (t-1) X ΔEV(t) | -0.25 | 0.073 | [-0.39 -0.108] | -3.43 | <0.001 | 14425 |
| Valence (t-1) X ΔEV(t) alone | 0.22 | 0.05 | [0.11, 0.32] | 4.32 | <0.001 | 7397 |
| Valence (t-1) X ΔEV(t) group | 0.034 | 0.052 | [-0.13, 0.06] | -0.647 | 0.517 | 7028 |
| ΔEV(t) alone negative | 1.22 | 0.08 | [1.05 1.402] | 14.61 | <.001 | 2281 |
| ΔEV(t) alone positive | 1.67 | 0.065 | [1.55, 1.807] | 27.17 | <.001 | 5116 |
| Group status(t-1) X Valence(t-1) X ΔEV(t) | -0.41 | 0.12 | [-0.64, -0.17] | 3.452 | 0.000556 | 7028 |
| Valence (t-1) X ΔEV(t) minority | -0.32 | 0.10 | [-0.52, -0.11] | -3.13 | 0.0017 | 1818 |
| ΔEV(t) after negative minority | 1.09 | 0.17 | [0.74, 1.503] | 6.30 | <.001 | 482 |
| ΔEV(t) after positive minority | 0.52 | 0.09 | [0.33, 0.71] | 5.46 | <.001 | 1336 |

*Note that in Experiment 2b, a triple interaction Previous condition*Magnitude*Valence was observed (parameter estimate=-0.15±0.076, Z=-1.98, p=0.047, no=14425), showing that the selective effect of magnitude after group rounds, was only present in the negative domain. This is why we report here the results after negative outcomes, while we don’t in the main tables. Indeed, in the corrected experiment 2 and meta-analytically across Experiments 1 and 2, an interaction between Previous condition and Condition appeared independently of valence.

**Supplementary table 7 – Lottery choice after orthogonalization**

General linear mixed models main results after the parametric ΔEV and ΔAR regressors were QR Gram Schmidt orthogonalized. These regressions yield to exactly the same statistical outputs (Z and p values, parameter estimates are in arbitrary units), showing that this orthogonalization was automatically performed in the initial mixed models with the correlated regressors.

| **Experiment 1** | | | | | |
| --- | --- | --- | --- | --- | --- |
| **Regressors** | **Parameter estimate** | **Standard error of estimate** | **Z value** | **P value** | **Number of observations** |
| Expected value ΔEV(t) | 107.46 | 4.29 | 25.03 | <.001 | 5799 |
| Anticipated regret ΔAR(t) | 7.58 | 3.34 | 2.26 | 0.0233 | 5799 |
| Current condition(t) X ΔAR(t) | 0.85 | 4.89 | 0.175 | 0.86 | 5799 |
| Current condition(t) X ΔEV(t) | 30.86 | 6.00 | 5.141 | <.001 | 5799 |
| Previous condition(t-1) X Valence (t-1) X ΔEV(t) | 23.99 | 8.32 | 2.883 | 0.0039 | 4268 |
| **Experiment 2** | | | | | |
| Expected value ΔEV(t) | 206.95 | 4.47 | 46.27 | <.001 | 23247 |
| Anticipated regret ΔAR(t) | 26.30 | 3.54 | 7.42 | <.001 | 23247 |
| Current condition(t) X ΔAR(t) | 12.29 | 4.95 | 2.48 | 0.0131 | 23247 |
| Current condition(t) X ΔEV(t) | 33.40 | 6.12 | 5.45 | <.001 | 23247 |
| Previous condition(t-1) X Valence (t-1) X ΔEV(t) | 19.16 | 0.082 | 2.33 | .019 | 17178 |

**Supplementary figure 1**

**
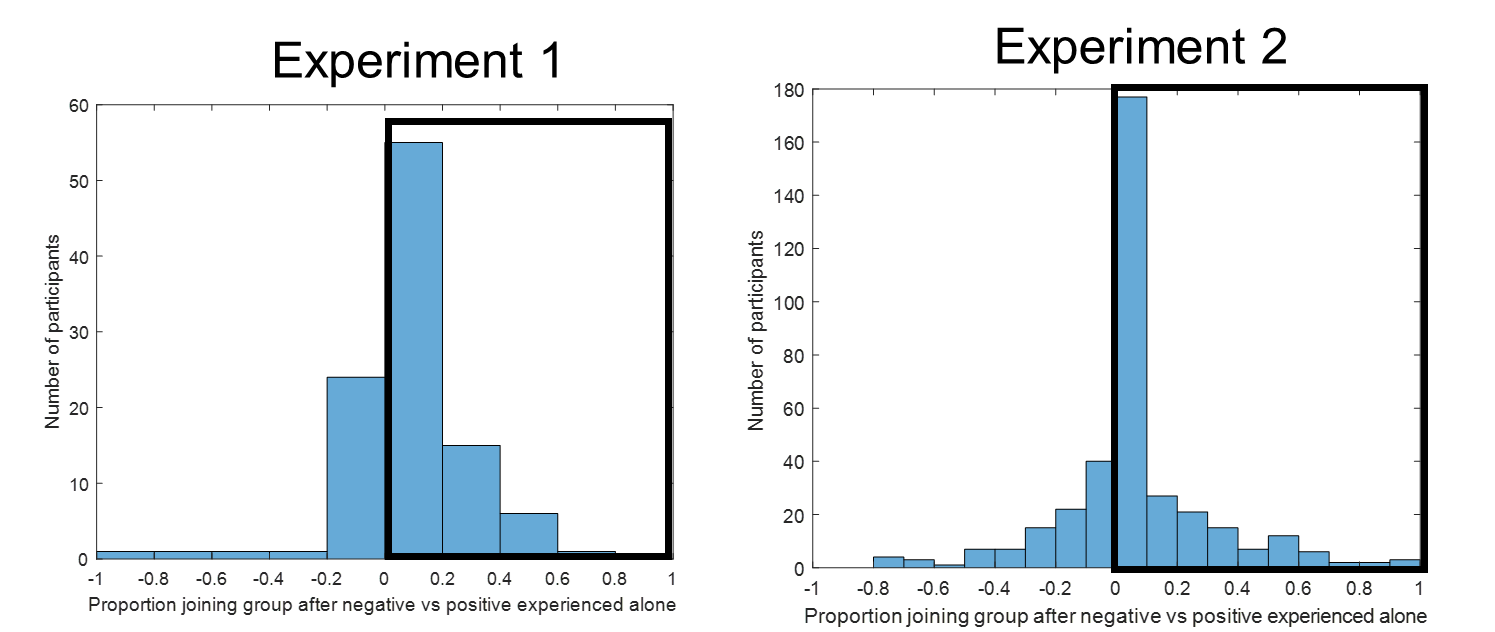
**

**Supplementary figure 1. Individual differences for joining groups based on outcome valence.** Histograms showing the difference in the proportion of joining groups after negative vs positive experienced outcome. The black squares highlight participants who switched from alone to group after experiencing negative vs positive outcomes.

**Supplementary figure 2**

**Supplementary Figure 2. Group majority and minority influence on playing in group. (a)** Proportion staying in a group after being the group majority or minority in the previous trial in Experiments 1 and 2. **(b)** Proportion staying in group after majority and minority as a function of the experienced outcome valence in the previous trial in Experiments 1 and 2.

**Supplementary results**

**Additional interpretation of triple interaction on lottery choice**

The triple interaction Previous Condition (t-1), Valence (t-1) and ΔEV(t) can be interpreted in 2 complementary ways:

1 -The way described in the results, based on the hypothesis we were testing: The interaction between Valence at t-1 and EV present after playing alone not group (and after group minority), showing a decreased EV after negative vs positive outcomes.

2 -The interaction between Previous condition and ΔEV present after positive but not negative outcomes. This suggests that after negative outcomes, whatever the condition (Alone, in group majority/minority) the ΔEV is similar on the next trial. But after positive outcomes, participants rely less on ΔEV if in group, and even less so if in the group minority. Running a model with the interaction between previous ConditionXEV separately after positive vs negative outcome indeed confirms this interpretation (interaction Previous ConditionXΔEV and Group StatusXΔEV only present after positive outcomes). This suggests that choice optimization is decreased after positive outcomes experienced in a group as compared to alone, and experienced in a group majority as compared to group minority. Nevertheless, this result does not cancel or change the fact that after group majority, the ΔEV parameter is stable whether after a positive or negative outcome – while the ΔEV changes between positive and negative outcomes experienced alone or in a group minority.
